# The Epidemiology of Insomnia and Sleep Duration Across Mental and Physical Health: The SHoT Study

**DOI:** 10.3389/fpsyg.2021.662572

**Published:** 2021-06-14

**Authors:** Børge Sivertsen, Mari Hysing, Allison G. Harvey, Keith J. Petrie

**Affiliations:** ^1^Department of Health Promotion, Norwegian Institute of Public Health, Bergen, Norway; ^2^Department of Research and Innovation, Helse-Fonna HF, Haugesund, Norway; ^3^Department of Mental Health, Norwegian University of Science and Technology, Trondheim, Norway; ^4^Department of Psychosocial Science, Faculty of Psychology, University of Bergen, Bergen, Norway; ^5^Department of Psychology, University of California, Berkeley, Berkeley, CA, United States; ^6^Department of Psychological Medicine, University of Auckland, Auckland, New Zealand

**Keywords:** insomnia, sleep, mental health, physical health, epidemiology

## Abstract

**Objective:**

Numerous epidemiological studies have been conducted to examine the prevalence and comorbidities of insomnia and document sleep duration, but a common limitation in many studies is the lack of use of agreed-upon definitions of insomnia, as well as insufficient statistical power to examine comorbid mental and physical disorders/conditions.

**Aim:**

To examine the prevalence of insomnia operationalized according to formal DSM-5 criteria and differences in mean sleep duration across a wide range of mental and physical disorders, examining men and women separately.

**Materials and Methods:**

Data stem from the SHoT study (Students’ Health and Wellbeing Study), a national survey of all college and university students in Norway. In all, 162,512 students aged 18–35 received an invitation to participate, of whom 50,054 students completed the internet-based survey (attendance rate: 30.8%). Insomnia was defined according to the Diagnostic and Statistical Manual of Mental Disorders (5th ed.) criteria and sleep duration was calculated separately for weekdays and weekends. Self-reported mental and physical disorders/conditions were assessed using a pre-defined list modified to fit this age group. Pearson chi-squared tests were used to examine the prevalence of insomnia across the various mental and physical disorders/conditions in men and women separately, and log-link binomial regression analysis were used to calculate effect-sizes, adjusting for age.

**Results:**

The prevalence of insomnia in both sexes was significantly higher across all mental disorders compared with a healthy reference group. Among females, the prevalence of insomnia ranged from 61.3% for comorbid depression (adj. RR = 2.49, 95% CI: 2.40) to 83.3% for comorbid schizophrenia (adj. RR = 3.37, 95% CI: 2.61–4.35). For males, the insomnia prevalence ranged from 32.3% for comorbid autism/Asperger (adj. RR = 2.02, 95% CI: 1.39–2.92) to 74.2% for comorbid eating disorder (adj. RR = 4.51, 95% CI: 3.87–5.27). The overall prevalence of insomnia was also significantly higher across most physical conditions compared with the healthy reference group, although generally lower compared to the mental disorders. For females, the insomnia prevalence ranged from 25% for comorbid multiple sclerosis (not significant) to 65.4% for comorbid chronic fatigue syndrome/ME (adj. RR = 2.66, 95% CI: 2.44–2.89). For males, the insomnia prevalence ranged from 20% for both comorbid cancer and diabetes (not significant) to 74.2% for comorbid fibromyalgia (adj. RR = 4.35, 95% CI: 2.96–6.39). Similar patterns were observed for sleep duration, with a significantly shorter sleep duration for across many physical disorders, but especially mental disorders.

**Conclusion:**

Insomnia and short sleep duration are strongly associated with a range of different disorders and conditions. Insomnia is most strongly associated with mental disorders, and physical conditions characterized by some level of psychological or psychosomatic properties.

## Introduction

Sleep problems, both in terms of sleep quality and sleep quantity, have been seen as key transdiagnostic processes across physical and mental health disorders. Specific sleep problems are listed as core symptoms across several of mental disorders [DSM-5 (American Psychiatric Association, 2013)]. While there is extensive literature showing a close link between insomnia and common mental disorders in adults, such as anxiety (Cox and Olatunji, 2020) and depression (Baglioni et al., 2011), fewer epidemiological studies have examined the prevalence of insomnia in less prevalent disorders, such as autism, obsessive compulsive disorder (OCD) and schizophrenia. Although insomnia may be regarded a transdiagnostic phenomenon (Harvey et al., 2011), the prevalence is likely to vary across the different psychiatric disorders. However, a common limitation in this field is the lack of use of agreed-upon definitions of insomnia, making comparisons across studies and disorders difficult.

There are also few studies that have included detailed measurements of sleep *quantity*, in contrast to sleep *quality*, and this is an important limitation, as sleep problems may take distinctive forms across the different disorders and conditions (John et al., 2005). For example, a higher risk of short sleep duration has been documented in adults with anxiety and depression in a population-based German sample (John et al., 2005), while a nationwide sample in Korea showed that psychiatric disorders in general were characterized by both very short and very long sleep duration (Park et al., 2010).

In contrast to the relationship between sleep and mental health problems, the link between physical health and insomnia has received less attention, although disturbed sleep is commonly reported across several somatic conditions (Fernandez-Mendoza and Vgontzas, 2013). For example, studies have shown that symptoms of insomnia occur frequently among patients with cancer (Irwin, 2013), type 1 diabetes (Irwin, 2013), chronic fatigue syndrome/myalgic encephalomyelitis (CFS/ME) (Mariman et al., 2013), asthma (Luyster et al., 2016), and migraine (Kim et al., 2017). Similarly, short and long sleep durations have been linked to specific illnesses such as type 2 diabetes and heart disease (Shan et al., 2015;

Seow et al., 2020). Research on psychiatric disorders is hampered by the many different operationalizations of sleep problems, limiting our ability to compare how insomnia and sleep duration varies across conditions and illnesses. There are also few large-scale epidemiological studies with sufficient statistical power to examine the less prevalent disorders and also few studies that include several disorders to allow for comparison between conditions.

Based on these considerations, the aim of this study was to examine the prevalence of insomnia operationalized according to formal DSM-5 criteria and differences in mean sleep duration across a wide range of mental and physical disorders. Associations were examined separately for men and women, as previous findings have suggested that while the rate of sleep problems and ill health differ between males and females, potential sex differences in the magnitude of these associations remain unknown.

## Materials and Methods

### Procedure and Setting

The SHoT2018 study was a national survey of all college and university students in Norway. Data were collected electronically through a web-based platform. Detailed information about the study has previously been published (Sivertsen et al., 2019a), but in short the SHoT2018 was conducted from February to April, 2018, and invited all fulltime Norwegian college and university students aged between 18 and 35 to participate. 162,512 students fulfilled the inclusion criteria, of whom 50,054 students provided valid responses to the web-based questionnaires, yielding an attendance rate of 30.8%. Whereas a few colleges and universities and did allocate some time in classes allowing the students to participate in the study during a lecture, teachers provided no assistance or support of any kind.

### Ethics

The SHoT2018 study was approved by the Regional Committee for Medical and Health Research Ethics in Western Norway (No. 2017/1176). An electronic informed consent was obtained after the participants had received a detailed introduction to the study.

### Instruments

#### Sleep Variables

##### Sleep duration

The students’ typical bed- and rise time were indicated in hours and minutes, and also sleep onset latency (SOL) and wake after sleep onset (WASO) were indicated separately for weekdays and weekends. Time in bed (TIB) was defined as the difference between bedtime and rise time, while sleep duration was calculated as TIB minus SOL and WASO. Only weekend sleep duration was used in the current study.

##### Insomnia

All students reported how many nights per week they experienced difficulties initiating sleep (DIS), difficulties maintaining sleep (DMS), early morning awakenings (EMA), in addition to daytime tiredness and sleepiness. Students were also asked about how long the sleep problems had been present. The following criteria were used as a proxy for insomnia disorder, according to the DSM-5 criteria: (1) reporting either DIS, DMS, or EMA for a minimum of 3 nights per week, (2) reporting daytime sleepiness and tiredness at least 3 days per week, and (3) the sleep problems had to last for at least 3 months. More details about the sleep inventory used in SHoT2018 has been published elsewhere (Sivertsen et al., 2019b).

#### Mental and Physical Disorders/Conditions

Self-reported mental and physical disorders/conditions were assessed by a pre-defined list which was modified to fit this age group. The list was based on a similar large population-based study [the HUNT studies (Krokstad et al., 2013)]. For mental disorders, the list included the following disorders or group of disorders: ADHD, anxiety disorder, autism/Asperger, bipolar disorder, depression, PTSD (posttraumatic stress disorder), schizophrenia, personality disorder, eating disorder, Tourette’s syndrome, and obsessive-compulsive disorder (OCD). The list of physical conditions included atopic conditions (allergy and intolerances, asthma, and eczema), neurological conditions (cerebral palsy, epilepsy, migraine, multiple sclerosis, and rheumatoid arthritis), psychosomatic conditions (chronic fatigue syndrome/myalgic encephalomyelitis [CFS/ME], fibromyalgia and irritable bowel), and other somatic conditions (cancer, type 1 diabetes, and heart disease).

#### Sociodemographic Information

All participants were asked about their sex and age. Participants also reported their household status (coded as “living alone” versus “living with others”), as well as their civil status (coded as “single” versus “married”/“partner” or “girl-/boyfriend”). In terms of ethnicity, participants were categorized as an immigrant if either the student or his/her parents were born outside Norway. Finally, all students reported their annual income, which was coded dichotomously: “economically active” (annual income > 10,000 NOK) versus “economically inactive” (<10,000 NOK).

### Statistics

IBM SPSS version 26 (SPSS Inc., Chicago, IL, United States) for Windows was used for all analyses. Pearson chi-squared tests were used to examine the prevalence of DSM-5 insomnia across the various mental and physical conditions in men and women separately, and log-link binomial regression analysis were used to calculate effect-sizes, adjusting for age. Results are presented as risk ratios (RR) with 95% confidence intervals, and the reference group was defined as participants not reporting any of the above disorders/conditions. Gender-specific estimated marginal means (EMM), adjusting for age, were calculated for sleep duration across all disorders. The normality of the data was examined using skewness and kurtosis, and the only continuous measures (sleep duration) was well within the recommended ranges (±2) (George and Mallery, 2016). *P*-values adjusted for multiple comparisons using the Benjamini-Hochberg’s false discovery rate (FDR). There was generally little missing data [*n* < 270 (0.5%)], and hence missing values were handled using listwise deletion. No *a priori* power calculations were conducted to ensure that the sample size had sufficient statistical power to detect differences in outcomes, as the SHoT study had several objectives and was not designed to be a study of these associations specifically.

## Results

### Sample Characteristics

The sample comprised 50,054 young adults (69.1% women), with a mean age of 23.2 years (*SD* = 3.3). In terms of civils status 49.9% (*n* = 24,969) reported being single (women: 47.2%, men: 56.0%, *P* < 0.001), while 19.3% reported living alone [*n* = 9,675; (women: 18.7%, men: 20.7%, *P* < 0.001)]. Most participants (87.5%, *n* = 43778) had an annual income of more than 10,000 NOK (women: 88.9%, men: 84.5%, *P* < 0.001). In terms of ethnicity, 8% percent of the sample (*n* = 4,010) were immigrants, defined as either the student or his/her parents being born outside Norway (women: 7.9%, men: 8.2%, *P* = 0.292).

### Insomnia in Mental Disorders

The overall prevalence of insomnia in the healthy reference group was 21.7% and was significantly higher among female (24.8%) compared to male participants (16.4%; see Table 1). As also detailed in Table 1, the prevalence of insomnia was significantly higher than the healthy reference group across all mental disorders in both genders. Among females, the prevalence of insomnia ranged from 59% for comorbid anxiety (adj. RR = 2.39, 95% CI: 2.30–2.49 to 83.3% for comorbid schizophrenia (adj. RR = 3.37, 95% CI: 2.61–4.35). For males, the insomnia prevalence ranged from 32.3% for comorbid autism/Asperger (adj. RR = 2.02, 95% CI: 1.39–2.92) to 74.2% for comorbid eating disorder (adj. RR = 4.51, 95% CI: 3.87–5.27).

**TABLE 1:** Prevalence and risk of DSM-5 insomnia across mental disorders stratified by sex in the SHoT2018 study.

	**Women (*n* = 34,437)**						**Men (*n* = 15,399)**					
	**Disorder (%)**	**(n)**	**Insomnia (%)**	**(n)**	**RR^$^**	**(95% CI)**	**Disorder (%)**	**(n)**	**Insomnia %**	**(n)**	**RR**	**(95% CI)**
**Healthy reference group**	n/a	24.8	(3583)	n/a		n/a	16.4	(1386)	n/a			
**Mental disorders**												
ADHD	0.9	(317)	63.7	(202)	2.56***	2.56-2.80	0.8	(127)	50.4	(64)	2.95***	2.47–3.53
Anxiety disorder	11.8	(4,055)	59.0	(2,391)	2.39***	2.30–2.49	6.1	(946)	51.9	(491)	3.07***	2.83–3.32
Autism/Asperger	0.2	(64)	68.8	(44)	2.77***	2.33–3.28	0.4	(62)	32.3	(20)	2.02***	1.39–2.92
Bipolar disorder	1.0	(350)	63.4	(222)	2.59***	2.38–2.81	0.7	(112)	55.4	(62)	3.15***	2.64–3.77
Depression	12.5	(4,309)	61.3	(2,640)	2.49***	2.40–2.58	7.7	(1,184)	55.4	(656)	3.31***	3.08–3.56
PTSD	2.4	(825)	65.6	(541)	2.67***	2.52–2.83	0.8	(123)	64.2	(79)	3.61***	3.10–4.19
Schizophrenia	0.04	(12)	83.3	(10)	3.37***	2.61–4.35	0.1	(14)	64.3	(9)	3.24***	2.16–4.86
Personality disorder	0.9	(325)	67.7	(220)	2.75***	2.53–2.99	0.5	(83)	54.2	(45)	3.16***	2.59–3.87
Eating disorder	3.5	(1,207)	61.7	(745)	2.50***	2.37–2.63	0.4	(83)	74.2	(46)	4.51***	3.87–5.27
Obsessive Compulsive Disorder	1.4	(472)	62.3	(294)	2.52***	2.34–2.72	0.6	(100)	46.0	(46)	2.72***	2.18–3.39
**Physical illnesses and conditions**												
*Atopic conditions*												
Allergy and intolerances	31.4	(10,815)	39.6	(4,280)	1.60***	1.54–1.66	27.9	(4,289)	26.3	(1,128)	1.60***	1.49–1.72
Asthma	9.2	(3,155)	42.3	(1,336)	1.71***	1.63–1.80	7.6	(1,171)	28.5	(334)	1.75***	1.58–1.94
Eczema	12.0	(4,140)	38.9	(1,612)	1.58***	1.50–1.66	6.9	(1,066)	26.6	(284)	1.60**	1.43–1.79
***Neurological conditions***												
Cerebral Palsy (CP)	0.1	(34)	50.0	(17)	1.97*	1.38–2.80	0.1	(16)	31.3	(5)	2.19*	1.09–4.40
Epilepsy	0.5	(168)	45.2	(76)	1.83**	1.54–2.17	0.4	(62)	21.0	(13)	1.31	0.81–2.17
Migraine	12.8	(4,421)	45.0	(1,990)	1.82***	1.74–1.90	4.6	(710)	37.9	(269)	2.28***	2.05–2.54
Multiple Sclerosis (MS)	0.1	(32)	25.0	(8)	1.01	0.56–1.85	0.1	(12)	16.7	(2)	n/a	
Rheumatoid arthritis	0.9	(293)	46.4	(136)	1.88***	1.65–2.14	0.5	(81)	46.9	(38)	2.77***	2.18–3.50
***Psychosomatic conditions***												
Chronic fatigue syndrome/ME	1.0	(338)	65.4	(221)	2.66***	2.44–2.89	0.2	(33)	48.5	(16)	2.81***	1.95–4.05
Fibromyalgia	0.8	(281)	62.3	(175)	2.54***	2.30–2.81	0.1	(8)	75.0	(6)	4.35***	2.96–6.39
Irritable bowel	10.0	(3,458)	45.1	(1,559)	1.83***	1.74–1.91	4.1	(631)	40.6	(256)	2.37***	2.13–2.64
***Other somatic conditions***												
Cancer	0.1	(31)	51.6	(16)	2.08***	1.48–2.94	0.1	(20)	20.0	(4)	n/a	
Diabetes (type 1)	0.7	(206)	38.3	(79)	1.64***	1.40–1.92	0.9	(116)	20.7	(24)	1.37	0.99–1.90
Heart disease	0.7	(238)	46.2	(110)	1.88***	1.63–2.16	0.7	(108)	36.1	(39)	2.17***	1.68–2.79

***p < 0.001; **p < 0.01; and *p < 0.05. p-values adjusted for multiple comparisons using the Benjamini-Hochberg’s false discovery rate (FDR). ^$^RR, relative risk (adjusted for age).

### Insomnia in Physical Conditions

The prevalence of insomnia was significantly higher across the majority of physical conditions compared to the healthy reference group, although was generally lower compared to mental disorders. As detailed in Table 1, for females, the insomnia prevalence ranged from 25% for comorbid multiple sclerosis (not significant) to 65.4% for comorbid chronic fatigue syndrome/ME (adj. RR = 2.66, 95% CI: 2.44–2.89). For males, the insomnia prevalence ranged from 16.7% for both comorbid cancer and diabetes (not significant) to 74.2% for comorbid fibromyalgia (adj. RR = 4.35, 95% CI: 2.96–6.39).

### Sleep Duration in Mental Disorders

**FIGURE 1:**
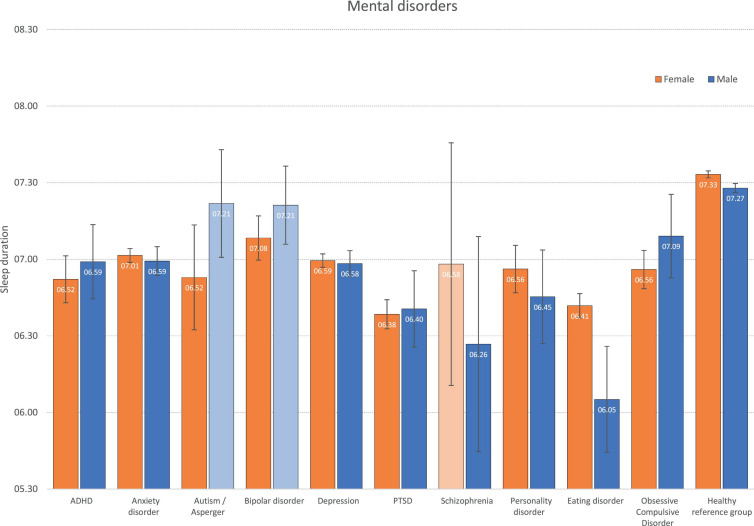
Weekday sleep duration across mental disorders by gender. Bars represent age-adjusted Estimated Marginal Means (EMM), and error bars represent 95% confidence intervals. Light-colored bars represent non-significant differences (*P* > 0.05) compared to the healthy reference group within each gender.

The average weekday sleep duration across all mental disorders are displayed in Figure 1. Compared to sleep duration in the healthy comparison group of females (7:33 h), females across most mental disorders (except schizophrenia) reported significantly shorter sleep duration, ranging from 6:52 h in PTSD to 7:08 in bipolar disorder. A similar pattern was observed for men, with a shorter sleep duration across most disorders (except autism and bipolar disorder). The shortest sleep duration among men was observed for eating disorders (6:05 h), compared to 7:37 h in the healthy control group. See Figure 1 for more details.

### Sleep Duration in Physical Conditions

**FIGURE 2:**
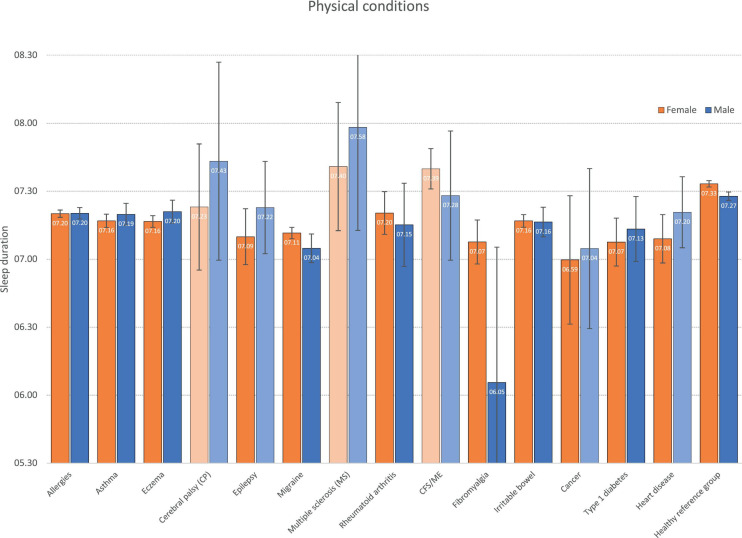
Weekday sleep duration across physical conditions by gender. Bars represent age-adjusted Estimated Marginal Means (EMM), and error bars represent 95% confidence intervals. Light-colored bars represent non-significant differences (*P* > 0.05) compared to the healthy reference group within each gender.

Sleep durations stratified by physical disorder are displayed in Figure 2. In most conditions, with some exceptions (CP, MS, and CFS/ME), the sleep duration in females was in general significantly shorter compared to the healthy reference group, although not at the same short level as found for most mental disorders. The findings were more mixed for men, with significantly shorter sleep duration observed for some conditions (allergies, asthma, eczema, migraine, RA, fibromyalgia, irritable bowel, and T1D), whereas other conditions (incl. CP, epilepsy, MS, CFS/ME, and cancer) did not differ significantly from the healthy reference group.

## Discussion

This study examined the rates of insomnia and sleep duration reported by a large sample of Norwegian young adults suffering from a range of mental and physical disorders, and compared the prevalence in these to healthy participants who did not report any disorder. The results showed that mental conditions had higher rates of sleep disturbance than physical illnesses, and females were more likely to be affected by sleep problems than males, in both physical and mental disorders. The rates of insomnia in mental conditions were at least two times higher than healthy controls. Among the mental disorders, PTSD, eating disorder, ADHD, and personality disorder showed the most impairment in sleep duration for both males and females. While in physical illness, sleep duration was most affected in cancer, migraine, fibromyalgia, and epilepsy.

Sleep problems, both short sleep duration and insomnia, were elevated across most physical and mental conditions indicating a transdiagnostic pattern. The findings of the study allow a comparison of sleep disturbance between physical and psychological conditions and highlight the pervasive nature of insomnia across mental conditions. This was supported by the data on weekday sleep duration, where lower sleep duration was observed for the mental disorders relative to the physical disorders. This is in line with previous studies (John et al., 2005). Interestingly, the weekday sleep duration was markedly lower in males and females reporting PTSD, where disturbed sleep is a recognized diagnostic feature (American Psychiatric Association, 2013). Also of note was the short weekday sleep duration among males with eating disorder. The study also found very high rates of insomnia in the psychosomatic conditions of CFS/ME, fibromyalgia, and irritable bowel syndrome that supports the current evidence base, although the pattern of sleep duration differed between the psychosomatic conditions, with only irritable bowel and fibromyalgia reporting shorter sleep duration than the healthy reference group. An expected gender pattern was present, with higher rates for insomnia among women compared to men for most conditions, while sleep duration showed more similarities across genders in the different health conditions, with the exception of the very low sleep duration among men with eating disturbances.

Data from future waves of this project may be able to establish the prognostic role of sleep disturbance in illness recovery or future health spending. Another important area is how behavioral interventions for insomnia may impact on longer-term outcome particularly in mental disorders where sleep disturbance is prominent. Indeed, research has shown that cognitive-behavior therapy for insomnia (CBT-I) may lead to improvements in levels of comorbid symptoms of depression and anxiety in insomnia patients (Belanger et al., 2016; Hagatun et al., 2018), and CBT-I has also been shown to substantially reduce both insomnia and pain levels in patients with fibromyalgia (Edinger et al., 2005). However, more research is needed to further explore to what extent treating sleep problems may improve health and functioning across other conditions and illnesses.

While the present findings demonstrate that reduced sleep quality and quantity is linked to ill health, the mechanisms or processes involved remain less clear. The consistent pattern of associations across nearly all conditions and illnesses may suggest that there are some common etiological factors which may drive the relationship. On a general level, sleep disturbances have been shown to be an important transdiagnostic process across most mental disorders (Harvey et al., 2011). For example, insomnia is etiologically associated with psychopathology through its reciprocal relationship to emotion regulation and the interplay between e.g., the dopamine and serotonin systems across sleep disturbances and several psychiatric disorders (Harvey et al., 2011). Another potential description of how insomnia may cause psychopathology has been proposed by Riemann et al. (2012) who suggested that fragmentation of REM-sleep may lead to impaired functionality of emotional neural networks which, again, may cause cognitive and emotional alterations, increasing the risk of depression. In terms of possible biological mechanisms linking sleep to physical illnesses, similar neurotransmitter systems may be involved. With dopamine being central in the regulation of the sleep–wake system, it has been suggested that somatic pain may change the signaling of dopamine, which in turn may negatively affect sleep quality (Monti and Monti, 2007). However, more research is required to investigate how sleep problems may change neurotransmitter functioning, and how this may ultimately affect our experience of ill health and pain, and vice versa. Other possible pathways between sleep problems and ill health have also been suggested, including immune function and inflammation, (Burgos et al., 2006) as well as pathophysiological processes such autonomic hyperarousal (Lanfranchi et al., 2009). Pain may also be a driver of insomnia and short sleep duration in somatic health conditions, but may also be bidirectional as insomnia has been linked to increased pain perceptions (Wei et al., 2018; McCrae et al., 2019). Medications may also impact sleep across mental and somatic disorders, including a positive effect of symptom management, but such drugs may also impact sleep negatively, as observed in conditions such as ADHD (Coogan et al., 2019) and epilepsy (Jain and Glauser, 2014).

### Methodological Considerations

Strengths of the current study include the large and heterogeneous sample, the extensive list of mental and physical disorders and conditions, and the use of well-validated sleep instruments. The most important study limitation is the cross-sectional design, which limits our ability to examine the directionality between sleep problems and the studied correlates. For example, poor health may be both a risk factor of reduced sleep quality and quantity (Hysing et al., 2018), as well as a consequence or co-morbid condition existing alongside poor sleep (Sivertsen et al., 2015). Another important limitation is the attendance rate of 31%, with no information about non-participants other than the age and gender distribution. The electronic survey approach may have played some role in the modest response rate, as web-based platforms have been shown to yield slightly lower attendance rates when compared to more traditional approaches (Greenlaw and Brown-Welty, 2009; Dykema et al., 2013). However, other reports have found similar attendance rates between online and article questionnaires (Horevoorts et al., 2015). Furthermore, the assessment of insomnia and sleep duration were based on self-report, and consequently lacks clinical evaluation and measurement by polysomnography or sleep diary. Moreover, the sample was comprised by college and university students, and as such, we were unable to examine and thus compare the prevalences of the included conditions/illnesses among young adults *not* pursuing higher education. Also, it should be noted as a study limitation that the pre-defined list of conditions/disorders included no information beyond self-reported presence of a of disorders/conditions (yes/no). Also, no follow-up questions regarding specific types disorders/conditions were asked. Finally, the data was obtained from February to April in a geographical region with substantial seasonal differences in daylight hours, which may have had an effect on sleep and its timing. Indeed, studies from high latitudes have found some sleep phase delay, with a slight increase in insomnia problems and fatigue during the winter as compared to the summer season (Husby and Lingjaerde, 1990; Johnsen et al., 2012, 2013; Friborg et al., 2014). However, results from a recent population-based study in Northern Norway found little evidence of seasonal variations in sleep (Sivertsen et al., 2020), even though the amount of daylight varied from 0 to 24 h across the year.

## Data Availability Statement

The datasets presented in this article are not readily available because the SHoT dataset was administrated by the NIPH. Approval from a Norwegian Regional Committee for Medical and Health Research Ethics [https://helseforskning.etikkom.no] is a pre requirement. Requests to access the datasets should be directed to corresponding author.

## Ethics Statement

The SHoT2018 study was approved by the Regional Committee for Medical and Health Research Ethics in Western Norway (No. 2017/1176). The patients/participants provided their written informed consent to participate in this study.

## Author Contributions

BS and MH conceived of the study. BS performed the data analyses in close cooperation with MH and wrote the first draft of the manuscript. AH and KP critically revised it for important intellectual content. All authors have read and approved the manuscript for submission.

## Conflict of Interest

The authors declare that the research was conducted in the absence of any commercial or financial relationships that could be construed as a potential conflict of interest.
